# Expression of Hepatoma-derived growth factor family members in the adult central nervous system

**DOI:** 10.1186/1471-2202-7-6

**Published:** 2006-01-23

**Authors:** Heba M El-Tahir, Frank Dietz, Ralf Dringen, Kerstin Schwabe, Karen Strenge, Sørge Kelm, Mekky M Abouzied, Volkmar Gieselmann, Sebastian Franken

**Affiliations:** 1 Institut für Physiologische Chemie, Rheinische Friedrich-Wilhelms Universität, Nussallee 11, 53115 Bonn, Germany; 2 Centre for Biomolecular Interactions Bremen, Universität Bremen, Leobener Straße, 28359 Bremen, Germany; 3 Institut für Hirnforschung, Universität Bremen, Leobener Straße, 28334 Bremen, Germany

## Abstract

**Background:**

Hepatoma-derived growth factor (HDGF) belongs to a polypeptide family containing five additional members called HDGF related proteins 1–4 (HRP-1 to -4) and Lens epithelial derived growth factor. Whereas some family members such as HDGF and HRP-2 are expressed in a wide range of tissues, the expression of others is very restricted. HRP-1 and -4 are only expressed in testis, HRP-3 only in the nervous system. Here we investigated the expression of HDGF, HRP-2 and HRP-3 in the central nervous system of adult mice on the cellular level by immunohistochemistry. In addition we performed Western blot analysis of various brain regions as well as neuronal and glial cell cultures.

**Results:**

HDGF was rather evenly expressed throughout all brain regions tested with the lowest expression in the substantia nigra. HRP-2 was strongly expressed in the thalamus, prefrontal and parietal cortex, neurohypophysis, and the cerebellum, HRP-3 in the bulbus olfactorius, piriform cortex and amygdala complex. HDGF and HRP-2 were found to be expressed by neurons, astrocytes and oligodendrocytes. In contrast, strong expression of HRP-3 in the adult nervous system is restricted to neurons, except for very weak expression in oligodendrocytes in the brain stem. Although the majority of neurons are HRP-3 positive, some like cerebellar granule cells are negative.

**Conclusion:**

The coexpression of HDGF and HRP-2 in glia and neurons as well as the coexpression of all three proteins in many neurons suggests different functions of members of the HDGF protein family in cells of the central nervous system that might include proliferation as well as cell survival. In addition the restricted expression of HRP-3 point to a special function of this family member for neuronal cells.

## Background

The family of Hepatoma derived growth factor (HDGF) and HDGF related proteins (HRPs) comprises six members which belong to different subgroups according to their length and isoelectric points. [1]. Little is known about the function of the different family members. So far, most studies addressed HDGF which was initially purified from the supernatant of human hepatoma cell lines [2,3].

Five HDGF homologous proteins have been identified so far [1,4,5]. Four of these proteins have been termed HRP-1 to -4 (HDGF Related Proteins 1 to 4), the fifth p52/75 or LEDGF (Lens Epithelium-derived growth factor). HDGF and its homologues display between 54% and 78% sequence identity among the 91 N-terminal amino acids. Because of this similarity the amino-terminal region has been termed *Homologue to Amino Terminus of HDGF *(HATH region [4]). In contrast, the length and amino acid sequence of HRP's C-terminal regions vary suggesting a modular structure of these proteins. This is supported by structural data obtained by NMR [6]. The main cellular localization of HDGF is nuclear, although in some cells HDGF can be found in the cytosol [3,7,8]. HDGF has two nuclear localization signals, one in the conserved HATH region, the other one in the C-terminal area specific for the different family members. The nuclear localization has been shown to be a prerequisite for the mitogenic activity of intracellular HDGF [9,10] whereas extracellular HDGF seems to signal through signal transduction pathways from the cell surface [11,12]. Except for their growth factor activity functions of HDGF family members are largely unknown. HRP-1 is believed to play a role in spermatogenesis [13] and LEDGF has been shown to function as a transcriptional activator. It also binds to and potentiates the activity of HIV integrase [14-17]. The latter activity has also been shown for HRP-2 [18]. For HDGF it has been speculated that it plays a role in renal, liver, lung and heart development [7,19-22]. In addition, a growing number of studies report a possible role of this growth factor in the development of different types of cancers [23-26]. In contrast, no functional data exist for HRP-3. The expression of this protein in contrast to the other family members is mainly restricted to nervous tissue [5,27].

Here we examine the cellular expression of HDGF, HRP-2 and HRP-3 in the adult rodent brain by Western blot analysis and immunhistochemistry. Data from these studies are compared to the expression of all three proteins in primary and secondary cell cultures of neurons, astrocytes, microglia and oligodendrocytes.

## Results

### Western blot analysis of various brain regions

Of the six members of the Hepatoma-derived growth factor family only HDGF, HRP-2 and HRP-3 are expressed in the central nervous system.

**Figure 1:**
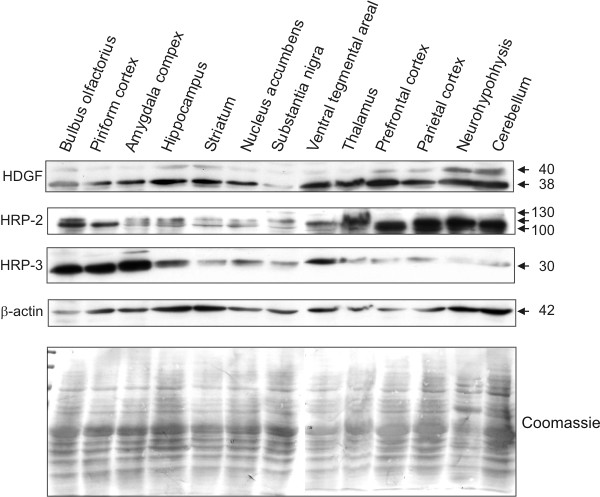
**Expression of HDGF family members in different brain regions**. Tissue homogenates of different rat brain regions were prepared as described under material and methods and subjected to Western blot analysis with antibodies against the proteins listed. Brain regions examined were as indicated above the figure. The Coomassie stained PVDF membrane is shown underneath the figure to demonstrate equal loading for the different brain regions. Molecular weights of marker proteins in kiloDalton are given on the right side of the figure.

To study the expression of these factors in more detail we performed Western blot analysis of samples obtained from different brain regions using HDGF, HRP-2, and HRP-3 specific antibodies. HDGF was rather evenly expressed throughout all brain regions investigated (Fig. 1), except for low levels in the substantia nigra. The HDGF-antiserum detected two polypeptides of 38 kDa and 40 kDa, the smaller form being predominant in all brain regions. In contrast to HDGF, expression of HRP-2 and HRP-3 varied substantially between different brain regions (Fig. 1). HRP-2 was strongly expressed in the thalamus, prefrontal and parietal cortex, neurohypophysis, and the cerebellum. In some tissues, the HRP-2 antiserum recognized up to three proteins of 90, 110 and 125 kDa.

HRP-3 expression was high in the bulbus olfactorius, piriform cortex and amygdala complex. The HRP-3 antiserum recognized a single polypeptide of 30 kDa, only in the amygdala complex an additional faint 35 kDa polypeptide was detectable. As for HDGF, substantia nigra contained only low amounts of HRP-2 and HRP-3 (Fig. 1).

### Immunohistochemical analysis of neuronal expression in brains of adult mice

**Figure 2:**
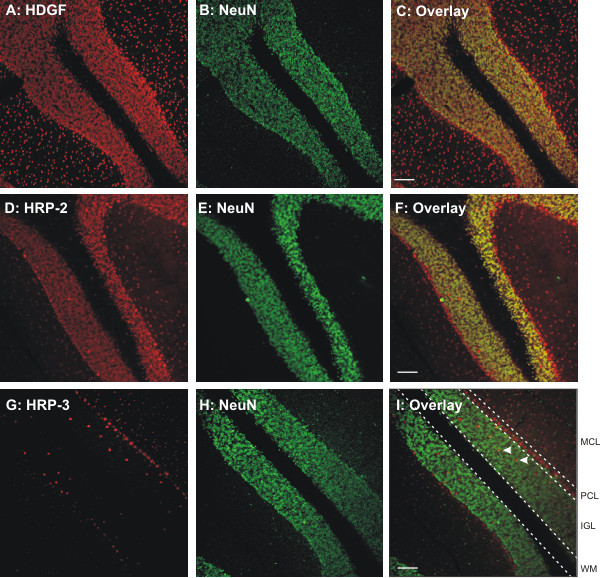
**Expression of HDGF family members in granular cells of the cerebellum**. Antibodies against HDGF (A), HRP-2 (D), HRP-3 (G) and NeuN (B, E, H) were used to detect the proteins in brain slices of adult mice. Double stainings demonstrate that in contrast to HDGF and HRP-2, HRP-3 is not expressed in NeuN positive granular cells of the cerebellum. Distinct cells in the IGL detected by HRP-3 antibodies are most likely Golgi cells (I, arrowheads). In addition HRP-3 is also clearly expressed in the PCL and weakly in some cells of the MCL (I). MCL: molecular cell layer; PCL: purkinje cell layer; IGL: internal granular cell layer; WM: white matter. Bars are 80 μm.

For a more detailed expression analysis we performed immunhistochemistry to detect HDGF, HRP-2 and HRP-3 in brain section of adult mice and compared their expression to that of cell type specific markers. Figure 2 shows cerebellar sections immunostained for HDGF (Fig. 2A), HRP-2 (Fig. 2D) and HRP-3 (Fig. 2G), respectively. These sections reveal that HDGF and HRP-2 are found in the internal granule cell layer (IGL), Purkinje cell layer (PCL) and to a lower extent in the molecular cell layer (MCL). Simultaneous immunostaining for HDGF or HRP-2 and NeuN – a marker for granule cells in the cerebellum [28] -, respectively, yielded comparable patterns of expression and confirmed that HDGF as well as HRP-2 are expressed in granule cells of the IGL (Fig. 2C+F). In contrast, HRP-3 did not colocalize with NeuN and is therefore not expressed by granule cells (Fig. 2I). Nevertheless a few HRP-3 positive cells were detected in the IGL (Fig. 2I arrowheads). These cells most likely represent Golgi cells. To a lower extent also cells of the MCL are stained most likely representing basket/stellate cells, another type of inhibitory interneuron in the cerebellum (Fig. 2G).

The overlay figures reveal that HDGF and HRP-2 expression is not restricted to granule cells. Cells in the adjacent PCL also strongly express HDGF and HRP-2 (Fig. 2C+F) as well as HRP-3 (Fig. 2I).

**Figure 3:**
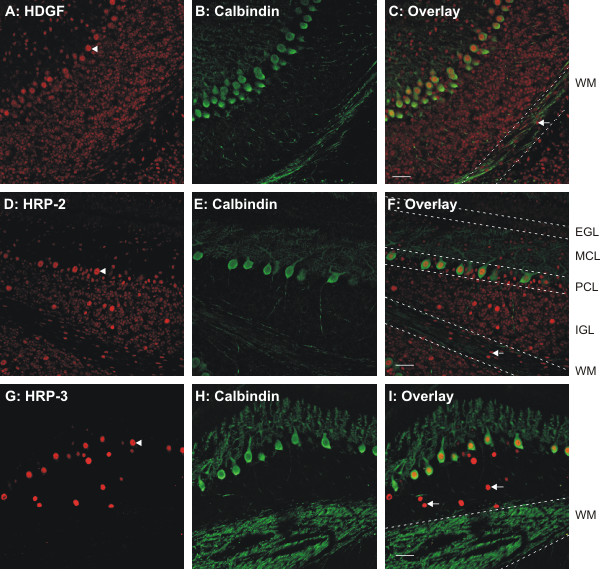
**Expression of HDGF family members in Purkinje cells**. Antibodies against HDGF (A), HRP-2 (D), HRP-3 (G) and Calbindin (B, E, H) were used to detect the respective proteins in brain slices of adult mice. Double stainings demonstrate that all three members are expressed in nuclei of Calbindin positive cells of the cerebellum (arrowheads in A, D and G). Arrows in C and F indicate HDGF and HRP-2 positive cells in the white matter. Arrows in I point to HRP-3 expressing cells in the IGL. EGL: external granular cell layer; MCL: molecular cell layer; PCL: Purkinje cell layer; IGL: internal granular cell layer; WM: white matter. Bars are 40 μm

To confirm the expression of HDGF, HRP-2 and HRP-3 in Purkinje cells double staining with calbindin a marker for these cells in murine cerebellum [29] was performed. Expression of all three family members was detected in the nuclei of Purkinje cells (arrowheads in Fig. 3A, D, G) whereas the cytoplasm of these cells is free of immunoreactivity (compare to calbindin in Fig. 3). As pointed out already, some cells in the IGL are strongly positive for HRP-3 and may represent Golgi cells (Fig. 3I arrows).

### Immunohistochemical analysis of glial expression in brains of adult mice

**Figure 4:**
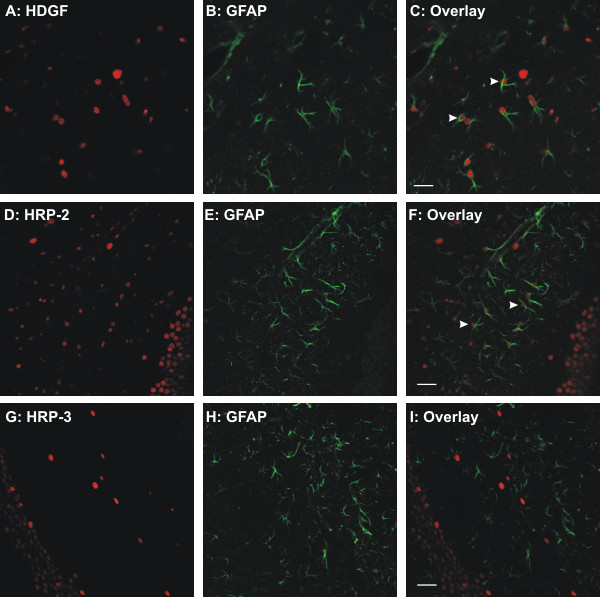
**Expression of HDGF family members in astrocytes**. Antibodies against HDGF (A), HRP-2 (D), HRP-3 (G) and GFAP (B, E, H) were used to detect the respective proteins in brain slices of adult mice. In all cases a part of the hippocampal region is shown. Double immunfluorescence demonstrates that HDGF (C, arrows) and HRP-2 (F, arrows) are expressed in cells positive for GFAP. In contrast no costaining of cells containing both GFAP as well as HRP-3 could be detected (I). Bars are 40 μm.

Figures 3C and 3F (arrows) show that HDGF and HRP-2 are also expressed in nuclei of cells within white matter tracts of the cerebellum (arrows in Fig. 3C+F). In contrast, no HRP-3 positive cells were detected in the cerebellar white matter (Fig. 3I). The occurrence of HDGF and HRP-2 positive cells in white matter tracts led us to investigate the expression of HDGF family members in glial cells. For this purpose we applied the specific antisera directed against the three proteins together with a monoclonal antibody recognizing glial fibrillary acidic protein (GFAP, Fig. 4). Figure 4 shows that cells with HDGF and HRP-2 positive nuclei also express GFAP (Fig. 4C+F arrowheads). In contrast, HRP-3 did not localize to GFAP positive cells (Fig. 4I). Therefore, astrocytes express HDGF and HRP-2 but no HRP-3.

**Figure 5:**
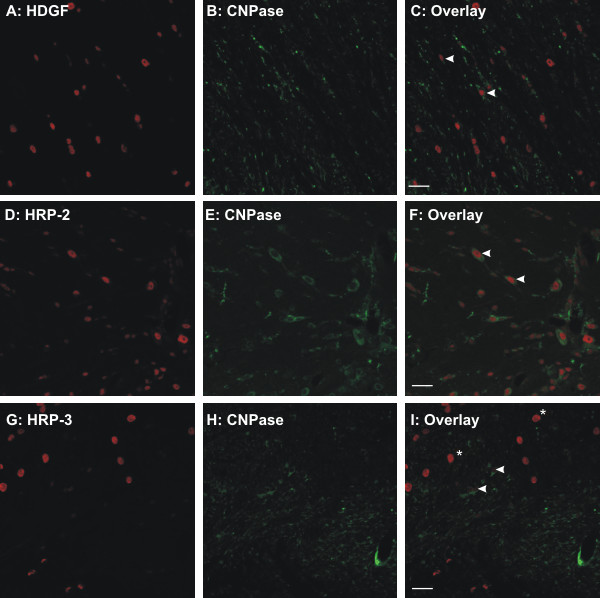
**Expression of HDGF family members in oligodendrocytes**. Antibodies against HDGF (A), HRP-2 (D), HRP-3 (G) and CNPase (B, E, H) were used to detect the respective proteins in brain slices of adult mice. In all cases fiber tracts in the brain stem are shown. Double immunfluorescence demonstrates that HDGF (C, arrowheads) and HRP-2 (F, arrowheads) are strongly expressed by cells also positive for CNPase. In contrast HRP-3 is only very weakly expressed by this cell type (I, arrowheads) when compared to other HRP-3 positive cells which most likely represent brain stem neurons (I, asterisks). Bars are 25 μm.

To investigate the expression of HDGF, HRP-2 and HRP-3 in oligodendrocytes we performed coimmunostainings on brain stem sections for these three proteins together with an antibody against 2,3-cyclonucleotid-phosphodiesterase (CNPase, Fig. 5). Again, HDGF and HRP-2 were strongly expressed by cells positive for CNPase (arrowheads in Fig. 5C+F) whereas these cells showed only very limited HRP-3 expression (Fig. 5I, arrowheads) when compared to cells most likely representing neuronal cells of the brain stem that are strongly positive for HRP-3 (Fig. 5I, asterisk).

### Examination of HDGF, HRP-2 and HRP-3 coexpreesion

**Figure 6:**
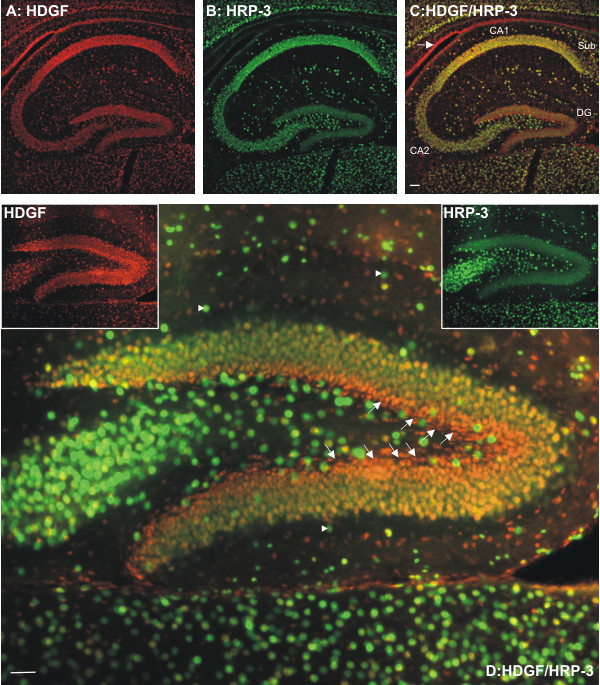
**Comparison of HDGF and HRP-3 expression**. Antibodies against HDGF (A) or HRP-3 (B) were used to detect both proteins in the hippocampus of an adult mouse. Comparison of the double immunfluorescence demonstrates the lack of expression of HRP-3 in cells covering the ventricel wall of the hippocampal region (C, arrow), whereas HDGF is clearly expressed in this cell type. In contrast, hippocampal neurons coexpress both family members, but whereas HDGF is found to a similar extent in all neurons of the hippocampal formation (A), neurons of the dentate gyrus show only weak expression of HRP-3 when compared to the rest of the hippocampus (B). Higher magnification of this region demonstrates the existence of cells predominantly expressing HRP-3 (D, arrowheads) as well as a cell layer only positive for HDGF (D, arrows). CA: cornu ammonis; Sub: subiculum; DG: dentate gyrus; Bar in C is 80 μm; bar in D is 40 μm.

Immunohistochemistry with the different cellular markers but also prima vista comparison of HDGF and HRP-2 expression in the cerebellum (e.g. Fig. 2A and 2D, Fig. 3A and 3D) revealed a considerable overlap in expression pattern for HDGF and HRP-2 in neuronal as well as glial cells. In contrast, coexpression with HRP-3 seemed to be restricted to special types of neuronal cells (for example Purkinje cells). To investigate the degree of coexpression of HDGF and HRP-3 in more detail we raised an antiserum against HDGF in sheep to perform double immunfluorescence of both proteins. In addition to cerebellar sections we also investigated hippocampal and cortical sections (Fig. 6 + 7).

These pictures reveal that HDGF expression is widespread in all brain areas investigated. The same applies for HRP-3, except for the cerebellum, in which the number of HRP-3 expressing cells is limited. There is considerable colocalization of HDGF and HRP-3 in the hippocampus but whereas HDGF is equally distributed over the whole hippocampus there is lower HRP-3 expression in the dentate gyrus than in the other parts of this brain region (Fig. 6A+B). In a higher magnification of this brain region cells can be distinguished that predominantly express HRP-3 (Fig. 6D, arrowheads) as well as a cell layer at the inner part of the dentate gyrus showing a particularly high expression of HDGF and being negative for HRP-3 (Fig. 6D, arrows). In addition, ependymal cells of the ventricular surface (Fig. 6C, arrow) where also found to be only positive for HDGF.

**Figure 7:**
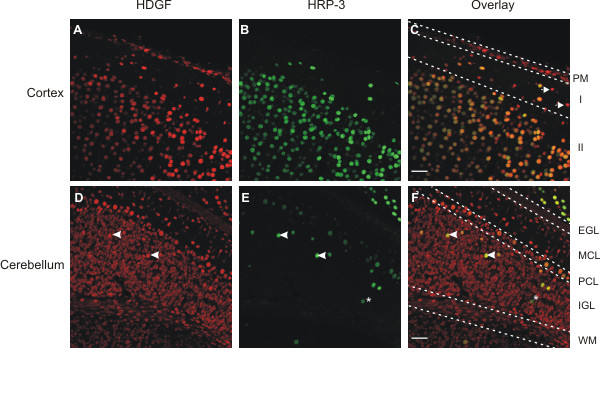
**HDGF and HRP-3 expression in the dentate gyrus**. Antibodies against HDGF (A, D) or HRP-3 (B, E) were used to detect both proteins in the neocortex and cerebellum of an adult mouse. Comparison of the double immunfluorescence demonstrates the lack of expression of HRP-3 in cells building the pia mater at the surface of the cortex (PM in C) and some cells in layer I (C, arrows), whereas cells in the rest of the cortex show a high degree of coexpression of HDGF and HRP-3. A lower degree of coexpression is found in the cerebellum, where HRP-3 family member is only expressed in Purkinje cells and Golgi cells of the IGL. Some of these HRP-3 positive cells do not (E+F, asterisks) and some do also express HDGF (D-F, arrowheads). PM: Pia Mater; I+II: layer I and II; EGL: external granular cell layer; MCL: molecular cell layer; PCL: purkinje cell layer; IGL: internal granular cell layer; WM: white matter. Bars are 40 μm.

In the cortex cells expressing HDGF and HRP-3 in layer I are less frequent than in layer II, however, there are only very few cells in layer I which express HDGF only (Fig. 7C, arrows). In good correlation to the ependymal cells mentioned above meningeal fibroblasts forming the pia mater are only expressing HDGF (Fig. 7C, PM).

In contrast to the hippocampus and cortex coexpression of HDGF and HRP-3 is limited in the cerebellum. As already shown in figure 2, in the cerebellum strongest expression of HRP-3 was found in cells most likely representing inhibitory interneurons of the internal granular cell layer (Golgi cells, Fig. 7D-F + 2I arrowheads). Very few unidentified cells in the IGL are only positive for HRP-3 (asterisks in Fig. 7E+F).

**Figure 8:**
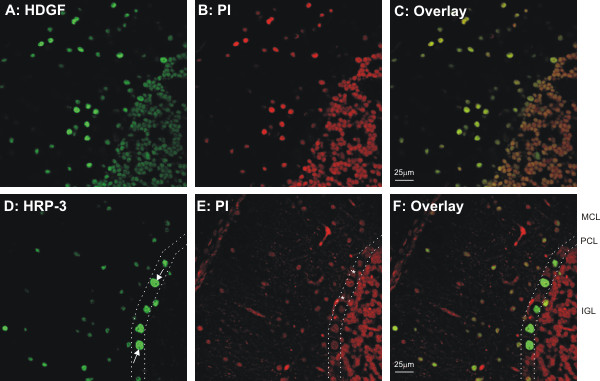
**Examination of the frequency of HDGF and HRP-3 expressing cells in the cerebellum**. Antibodies against HDGF (A, C) or HRP-3 (D, F) were used to detect both proteins in the cerebellum of an adult mouse. To label the nuclei of all cells in the sections counterstaining with propidium iodide was performed (B,C and E,F). Double stained sections were used to calculate the percentage of cells positive for HDGF or HRP-3, respectively. Whereas HDGF is expressed in all cells, HRP-3 expression is much more restricted (for details see text). Cells labeled with arrows in D represent HRP-3 positive Purkinje cells. Asterisks in E mark most likely radial glia cells. MCL: molecular cell layer; PCL: purkinje cell layer; IGL: internal granular cell layer; Bars are 25 μm.

In the double stainings between HDGF and HRP-3 differences in the pattern of expression were most striking in the cerebellum (Fig. 7D-F). Therefore, to get an idea about the percentages of cells in this brain region positive for the two proteins we performed double staining with propidium iodide to label the nuclei of all cells. Figure 8 shows examples for these stainings for HDGF (Fig. 8A-C) and HRP-3 (Fig. 8D-F). For each marker three independent pictures were taken and used to count all cells (propidium iodide) and cells positive for HDGF or HRP-3, respectively. Cells were independently counted for the molecular layer (MCL) on the one hand and the Purkinje cell layer (PCL) together with the internal granule cell layer (IGL) on the other hand. As can be seen already in figure 8A–C 100% of the cells in the cerebellum express HDGF. For HRP-3 we obtained different results for the two counted regions. In the MCL in the three pictures examined 64, 68 and 69% of all cells showed HRP-3 immunreactivity. Because of the restriction of HRP-3 to neuronal cells this cells most likely represent basket/stellate cells another type of inhibitory interneuron in the cerebellum. In the PCL and IGL together because of the high amount of granule cells negative for this family member less than 5% of cells were positive for HRP-3. As mentioned before Purkinje cells are positive for HRP-3 (Fig. 8D arrows). As can be seen in figure 8E there are additional cells in the Purkinje cell layer that do not stain for HRP-3 most likely representing the cell bodies of radial glia cells that lie in direct contact with the Purkinje cell bodies in this cell layer (Fig. 8E asterisks). In addition there is not a sharp boundary between this layer and the granule cell layer. Therefore some of this HRP-3 negative cells display also granule cells.

### Expression of HDGF, HRP-2 and HRP-3 in cultured cells

**Figure 9:**
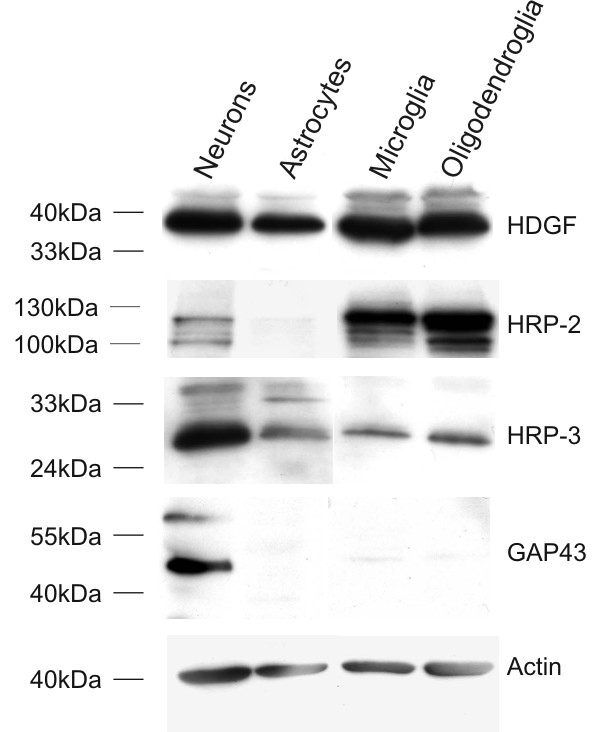
**Expression of HDGF family members in neural cell culture**. Proteins extracted from cultured neurons, astrocytes, microglia, and oligodendrocytes were subjected to SDS-PAGE as described under material and methods. After blot transfer membrane was incubated with antibodies against the different HDGF family members or GAP-43 and actin as controls. Whereas HDGF is expressed to a similar extent in all four cell types, HRP-2 and HRP-3 show a more restricted expression pattern in neural cell cultures. Molecular weights of marker proteins in kiloDalton are given on the left.

To compare the results obtained by immunhistochemistry of brain sections of adult mice with cell cultures of neuronal and glial cells, we prepared cultures of neurons, astrocytes, microglia and oligodendrocytes as described in material and methods. HDGF, HRP-2 and HRP-3 were quantified in cell homogenates by Western blot analysis. For HDGF and HRP-3 the results of these Western blots confirmed the data of immunhistochemistry. Whereas HDGF showed strong expression in all of the four cell types, high amounts of HRP-3 were only found in cultured neurons (Fig. 9). Only low amounts of HRP-3 were detected in glial cell cultures. To exclude that this observation might be due to residual neuronal cells in the glial cell culture we analyzed the samples for the presence of the neuron specific marker protein growth-associated protein-43 (GAP-43). This protein was only detected in cultured neurons, but not in the glial cell cultures. In contrast to the data obtained on adult mouse brain sections, HRP-2 is not or only weakly expressed in cultured astrocytes or neuronal cells, respectively. Strong HRP-2 expression was found in cultured oligodendrocytes and microglia.

## Discussion

The family of Hepatoma derived growth factor related proteins comprises six members. Whereas HDGF and HRP-2 are expressed in a wide variety of tissues including the nervous system, HRP-3 is expressed in the nervous system only. Previous data indicate that at least HDGF and HRP-3 proteins are expressed by differentiated neurons [27]. Western blots of protein extracts of different brain regions demonstrate a rather ubiquitous expression of HDGF family members in various CNS regions. HDGF is the most evenly distributed member whereas HRP-2 and HRP-3 expression varies between different regions (Fig. 1). On the cellular level HRP-3 shows the most restricted expression when compared to HDGF and HRP-3. HRP-3 was strongly expressed in neurons only. Expression in neurons is, however, not ubiquitous but occurs only in a subpopulation: e.g. Purkinje cells in the cerebellum and neurons within the subiculum and cornu ammonis of the hippocampus are strongly positive whereas cerebellar granule cells are negative for HRP-3. The term granular cell is used to describe major cell populations in the cerebellar cortex the olfactory bulb and the dentate gyrus. These cells share the characteristics of being born late during development and they all express at least one common molecular marker, termed RU49, that has been implicated in their specification [30]. This has led to the suggestion that these diverse kinds of granule cells are of a common developmental origin [30]. This is supported by the observation that granule cells of the dentate gyrus show a remarkably reduced expression of HRP-3 in addition to its missing expression in cerebellar granule cells described above.

Beside the correlation between HRP-3 and neuronal origin, the limited cerebellar expression of this protein regarding neuron subtypes correlates also with the transmitter phenotype of this cells. Whereas granule cells are glutamatergic all other neurons of this brain structure use gamma amino butyric acid (GABA) as a neurotransmitter. Ptf1a, which was reported to be a lineage determinator in the cerebellum, for example, is also exclusively expressed by GABAergic neurons of the cerebellum [31,32]. At least for the cerebellum HRP-3 might therefore be a marker to distinguish between excitatory and inhibitory neuronal subpopulations.

Except for neurons very low amounts of HRP-3 could also be detected in oligodendrocytes located in the brain stem. In contrast, white matter tracts of the cerebellum contained no HRP-3 expressing cells. Studies on the place and time of origin of oligodendrocytes have demonstrated heterogeneity inside of this cell population [33]. Maybe HRP-3 expression in oligodendrocytes similar to our observations for granule neurons is also dependent on their time of birth. In contrast to oligodendrocytes HRP-3 could not be detected in astrocytes in vivo.

Expression of HDGF and HRP-2 is much less restricted than that of HRP-3. Both proteins are found in neurons, astrocytes and oligodendroglia, in meningeal fibroblasts and ependymal cells. Immunohistochemistry shows that HDGF and HRP- 2 expression in neurons is widespread, except for the cerebellum this also applies for HRP-3. Our data indicate that all three proteins are found in the majority of neurons. However, our data do not allow to exclude that a minor subpopulation of neurons may in fact be negative for HDGF and HRP-2.

The results obtained by immunohistochemistry of brain slices are only partly reflected in cultured cells.

As expected, in cultured cells HRP-3 is predominantly expressed in neurons and only to a low extent in glial cells. Expression levels of HDGF are similar in all cells investigated, which also reflects the in vivo situation. Clear differences between cell culture and tissue data regarding the expressing cell type were detected in the case of HRP-2. Whereas in immunhistochemistry cells positive for GFAP showed also HRP-2 immunreactivity, no HRP-2 protein could be detected in the astrocytic culture. This may either reflect species differences since the cultures were prepared from rat brain or developmental differences, because immunohistochemistry was performed on brain sections of adult animals, whereas cultures were prepared from neonatal rats.

It has already been noted previously that the antisera against the various HRP proteins detect more than one polypeptide in Western blot analysis. The HDGF antiserum recognizes a predominant 38 kDa and a minor 40 kDa polypeptide, respectively. The molecular basis for this is unclear. Similarly, the HRP-2 antiserum detects three polypeptides of 90, 110 and 125 kDa in Western blots. Thus, as already shown for HDGF and HRP-3 [27] also HRP-2 is migrating at a molecular weight significantly higher than the one predicted (74 kDa) by its primary amino acid sequence. Whether this is due to posttranslational modifications or as shown for HDGF and HRP-3 to abnormal migration behavior in SDS-PAGE has to be determined. In contrast, in most tissues only a single HRP-3 polypeptide can be detected. Only in homogenates of piriform cortex and the amygdala complex a faint 35 kDa polypeptide is detectable. Similarly, low amounts of additional 35 kDa and 33 kDa polypeptides cross react in homogenates of cultured neurons and astrocytes respectively. Thus, all of the antisera detect several polypeptides. The functional significance of these different polypeptides is unknown.

The overlapping expression pattern of HDGF and HRP-2 and for neuronal cells also HRP-3 suggests different functions for these proteins. Beside its proliferating activity HDGF displays also survival activity for neurons [34]. A relation of this factor to cell death and survival is further underlined by the observation that it is involved in TNFα induced apoptosis in Hela cells [35]. It can be speculated that the function of HDGF in neurons during development changes from more proliferative aspects to cellular survival. Expression in glial cells indicates that within the nervous system HDGF may have similar functions also in non-neuronal cells. For HRP-2 much less is known regarding the function of this family member. Structurally it shows the highest similarities to LEDGF and also functionally their might be an overlap between these two proteins. For example, for both of them it was shown that they bind to and enhance the activity of HIV integrase [18]. Similar to HDGF, LEDGF was reported to be neuroprotective [36]. Whether this is also true for HRP-2 has to be determined.

HRP-3 in contrast to the other two family members seems to be restricted to neurons, but the nature of its function in these cells is still obscure. Beside its localisation in the cell nucleus at least in younger animals a prominent protein signal in neurites can be observed [27]. Whether HRP-3 therefore is involved in the extension or maintenance of these cellular processes has to be addressed in the future.

## Conclusion

Prior studies have demonstrated HDGF as well as HRP-3 expression in the central nervous system [12,34]. The presented data show a wide distribution of both family members as well as HRP-2 in brain neurons. In contrast glial cells express only low amount or even no HRP-3 but HDGF as well as HRP-2. This observation point to a special function of HRP-3 for neuronal cells. In addition, the overlapping expression pattern of HDGF and HRP-2 and for neuronal cells also HRP-3 suggests different functions for these proteins that might include proliferation as well as cell survival.

## Methods

### Antibodies

For Western blot analysis antibodies against β-actin (pan Ab-5) was obtained from Dianova (Hamburg, Germany) and against GAP-43 from Chemicon International (Hofheim, Germany). For immunhistochemistry antibodies against NeuN, Calbindin and GFAP were from Chemicon International, Vector Laboratories (Burlingham, CA, USA) and Sigma (Munich, Germany) respectively.

Rabbit antibodies against HDGF, HRP-2 and HRP-3 were produced and purified as described elsewhere [1]. The sheep antiserum against HDGF was raised against the histidine tagged protein as described for the rabbit antibodies [1,27]. Three aliquots of about 500 μg protein each were used to immunize a sheep (Diagnostics Scotland, Edinburgh, Scotland). Immunization was performed according to local governmental regulations. In order to obtain specific antibodies suitable for immunhistochemistry rabbit and sheep sera were purified by affinity chromatography using GST (glutathione S-transferase) fusion proteins of the different growth factors. Therefore, mouse HDGF, HRP-2 and HRP-3 coding regions were cloned into the pGEX4T3 vector (Amersham Pharmacia Biotech, Freiburg, Germany) using BamHI and SalI restriction sites. After purification via glutathione sepharose (Amersham Pharmacia Biotech, Freiburg, Germany) the purified proteins were coupled to Affigel 10 using the protocol supplied by the manufacturer (Bio-Rad, München, Germany). The resulting matrices were used for immunoaffinity purification of antibodies specific for the respective growth factor. All antibodies were examined for crossreactivity by preincubation with the respective recombinant protein (for results see file 1 and 2 of the additional material and Abouzied and coworkers [27]).

All fluorescently labeled secondary antibodies used for immunhistochemistry were obtained from Dianova (Hamburg, Germany).

### Preparation of protein extracts and western blot analysis

Protein extracts from the different brain regions of adult rat brain were prepared by homogenization in TBS (Tris-buffered saline; 20 mM Tris/HCl, pH 7.0, 150 mM NaCl) containing 1% Nonidet P40 and protease inhibitors (5 mM EDTA, 2 mM phenylmethyl sulfonylfluoride, 1 μg/ml leupeptin and 1 μg/ml pepstatin). After homogenization, samples were centrifuged at 20.000 g for 20 min at 4°C to remove unhomogenized material. The protein content in the supernatant was determined by a detergent-compatible assay (BCA™ protein assay; Pierce). Equal amounts of protein for each brain region were loaded on to a SDS/PAGE gel (10% acrylamide) and resolved according to the method of Laemmli [37].

To obtain protein samples for Western blotting, neural cell cultures (on 50 mm dishes) were washed with 5 ml ice-cold phosphate buffered saline (PBS, 10 mM potassium phosphate buffer pH 7.4, containing 150 mM NaCl) and lysed for 10 minutes in 400 μl lysis solution (0.3 μM aprotinin, 1 μM leupeptin, 1 μM pepstatin, 100 μM phenylmethyl sulfonylfluoride in H_2_O) on ice. Aliquots of the lysates were lyophilised and used for protein assays and for Western blot analysis.

After electrophoresis, proteins were transferred to a PVDF membrane (Immobilon-P™; Millipore, Schwalbach, Germany). Free binding sites on the membrane were blocked by incubation in 3% (w/v) skimmed milk in TBST (TBS containing 0.05% Tween 20). Primary antibodies against HRP-3 (1:1.000), HRP-2 (1:500), HDGF (1:1.000), actin (1:10.000) and GAP 43 (1:1.000) and peroxidase-labelled secondary antibodies against rabbit and sheep (both 1:20.000; Dianova) were incubated in TBST containing 3% skimmed milk for 2 h at room temperature. After three washings in TBST, bound antibodies were visualized by ECL^® ^system (Amersham Pharmacia Biotech). For the detection of the different target proteins on the same PVDF membrane remaining antibodies were stripped by incubation with Glycine/HCl pH 3.0 for 20 min at room temperature. For the Western blot of the brain region homogenates detection was performed in the order HRP-2/HDGF/HRP-3/actin, for the Western blot of the neural cell culture samples in the order HRP-2/HDGF/HRP-3/actin/GAP 43, respectively.

### Cell cultures of neural cells

Neuron-rich primary cultures were prepared from the brains of embryonal (E16) Wistar rats as previously described [38]. Experiments were conducted at an age of 6 days. These cultures contain approximately 5% astroglial cells [39] but no oligodendroglial or ependymal cells [38]. Astroglia-rich primary cultures derived from the brains of neonatal Wistar rats were prepared and maintained as described [40]. The results were obtained with 14- to 21-day-old cultures. These cultures contain minor numbers of oligodendroglial, ependymal and microglial cells [41]. Microglia-rich secondary cultures were prepared from astroglia-rich primary cultures as described previously [42]. The cultures were used at an age of 6 days. These cultures contain about 90% microglial cells and small quantities of astroglial and oligodendroglial cells [42]. Oligodendroglia-rich secondary cultures derived from astroglia-rich primary cultures in 175 cm^2 ^flasks were prepared as recently described [43]. The cultures were used at an age of 6 days. The cultures contain about 90% oligodendroglial cells and the majority of the remaining cells are astroglial cells [43].

### Immunhistochemistry

Immunhistochemistry was performed as described before. Briefly, mice were deeply anesthetized by intraperitoneal injection of 2.5% avertin in 0.9% saline (0.8 ml/100 g body weight). Subsequently, they were transcardially perfused with 0.4 ml/g body weight of Ringers solution for mammalians, followed by 2 ml/g body weight of 4% (w/v) freshly prepared paraformaldehyde in PBS. Following perfusion, brains were removed from the skull and postfixed for 7 h at room temperature in the same fixative and afterwards kept in PBS over night. Then, 40 μm thick, sagittal sections were cut in PBS using a Leica vibratome (VT1000S, Leica, Wetzlar, Germany). To visualize the different antigens, sections were washed in PBS for 10 min and permeabilized by incubation in 0.5% Triton X-100/PBS for 30 min. Non-specific protein binding sites were blocked using 2% bovine serum albumin in PBS for 1 h followed by incubation of the primary antibodies in blocking solution overnight at 4°C in a humidified chamber. Unbound antibodies were removed by washing in PBS and sections were incubated with the respective fluorescently labelled antibodies diluted in blocking solution for 2 h at RT. After 2 washes in PBS and one wash in distilled water immunfluorescence was analyzed by laser scan microscopy using a Leica TCS SP2 instrument.

## Authors' contributions

HME carried out the immunhistochemical experiments and participated in the design of the study and the drawing up of the manuscript. FD carried out the preparation of the brain tissue samples, performed all western blot studies and participated in the design of the studies. KS was responsible for the preparation of the different brain sections. RD prepared the neuronal cell culture samples. KST participated in the preparation of the brain tissue samples. SK participated in the design of the study. MMA participated in the immunhistochemical approach and the production, purification and characterization of the antibodies. VG participated in the design and coordination of the study and helped to draft the manuscript. SF designed the study organized the collaboration and finalized the manuscript. All authors read and approved the final manuscript.

## Supplementary Material

Additional File 1 Antibody preabsorption for Western blot analysis. Protein extracts from two independent preparations of adult rat brain were subjected to SDS-PAGE on 12% acrylamide gels. Proteins were transferred to nitrocellulose and tested for HDGF, HRP-2 and HRP-3 with the antibodies given above the figures. For preabsorption antibodies were incubated 4 h at RT with a 40 molar excess of the respective recombinant protein before incubation with the membrane. Click here for file

Additional File 2 Antibody preabsorption for immunhistochemistry. Vibratome cut adult mouse brain slices were stained with the antibodies given beside the figures (green) and propidium iodide for cellular counterstaining (red). For preabsorption antibodies were incubated 4 h at RT with a 40 molar excess of the respective recombinant protein before incubation with the brain slices. Bound antibodies were detected by fluorescently labeled secondary antibodies. Click here for file
