# Supplementary figures and images for: Expression of Hepatoma-derived growth factor family members in the adult central nervous system
Source: BMC Neurosci. 2006 Jan 23;7:6. doi: 10.1186/1471-2202-7-6 (PMC1363353; doi:10.1186/1471-2202-7-6)

# Antibody preabsorption for Western blot analysis

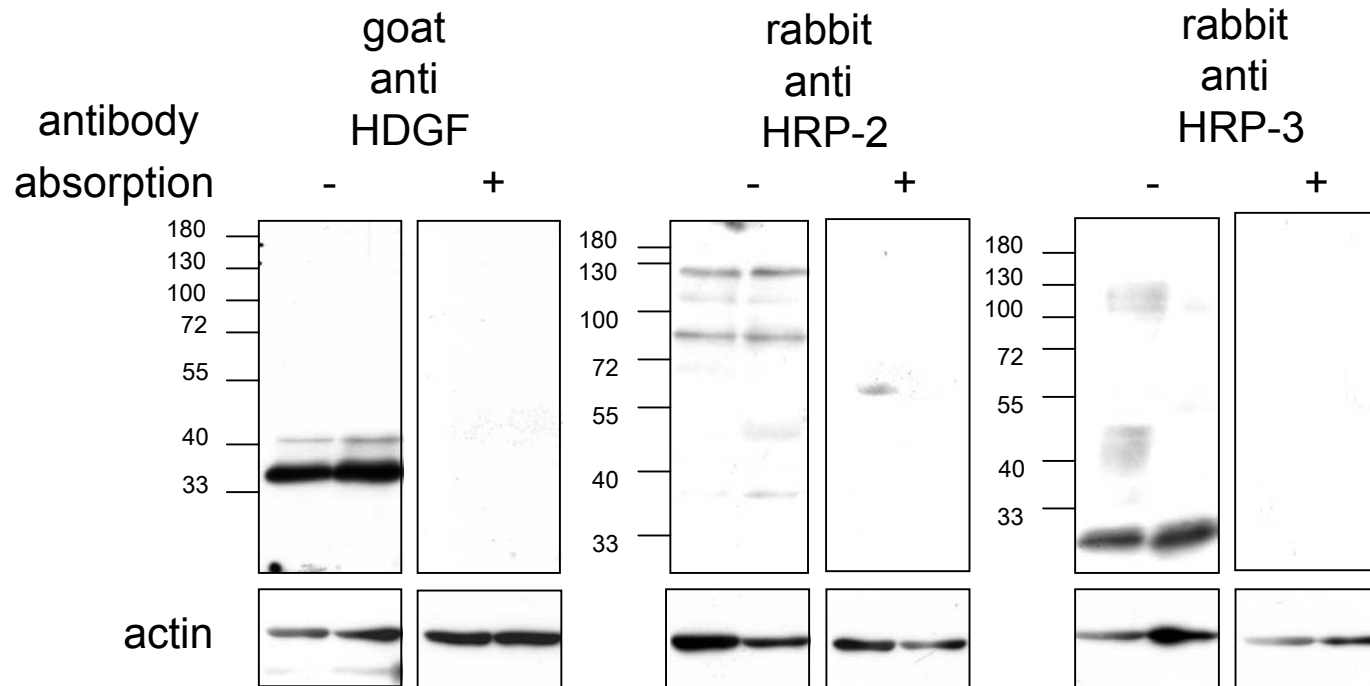

Supplement: Additional File 1 — Antibody preabsorption for Western blot analysis. Protein extracts from two independent preparations of adult rat brain were subjected to SDS-PAGE on 12% acrylamide gels. Proteins were transferred to nitrocellulose and tested for HDGF, HRP-2 and HRP-3 with the antibodies given above the figures. For preabsorption antibodies were incubated 4 h at RT with a 40 molar excess of the respective recombinant protein before incubation with the membrane. [file 1471-2202-7-6-S1.pdf]
