# Supplementary material for: Expression of Hepatoma-derived growth factor family members in the adult central nervous system
Source: BMC Neurosci. 2006 Jan 23;7:6. doi: 10.1186/1471-2202-7-6 (PMC1363353; doi:10.1186/1471-2202-7-6)

Antibody preabsorption for immunhistochemistry

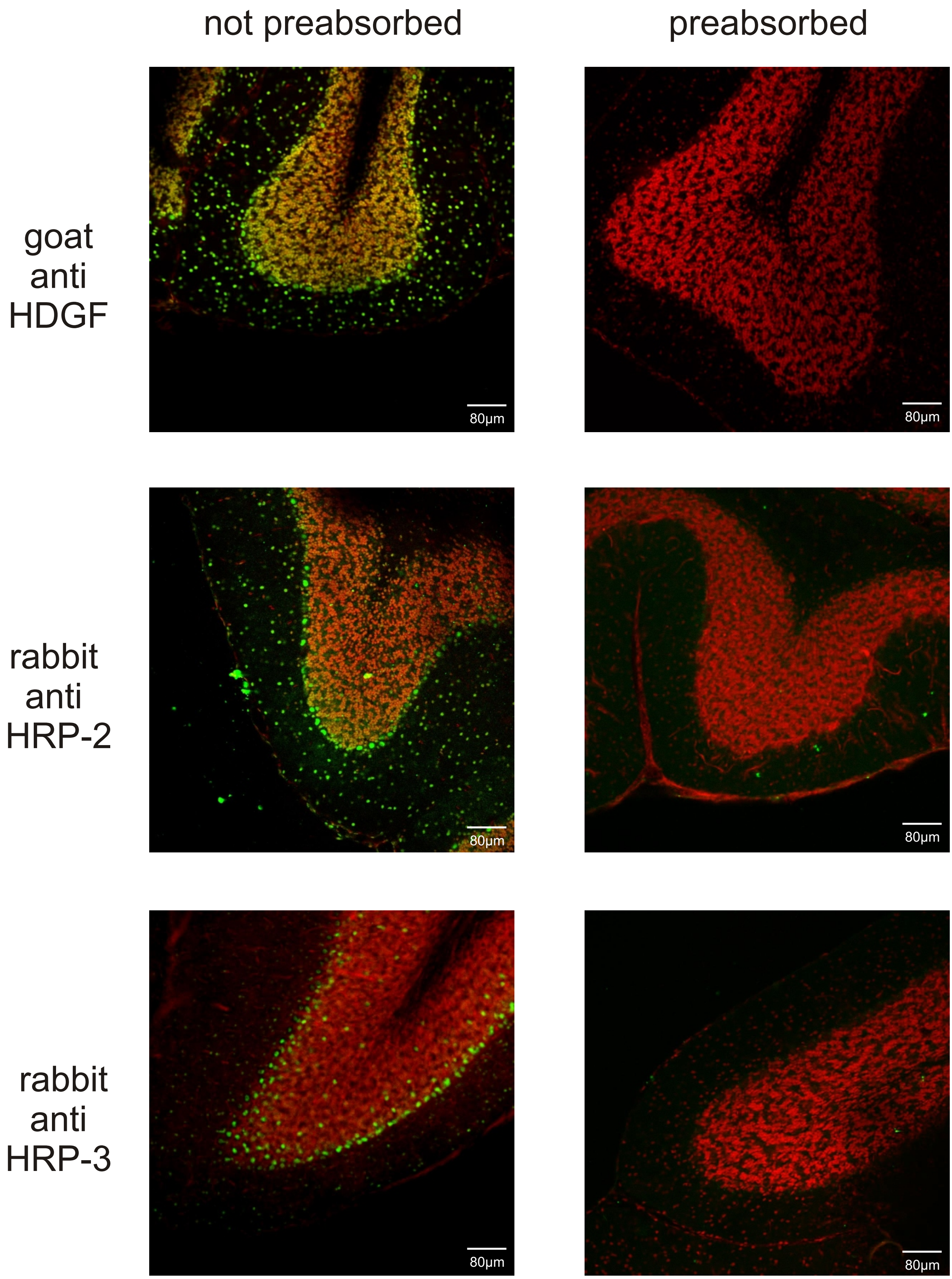

Supplement: Additional File 2 — Antibody preabsorption for immunhistochemistry. Vibratome cut adult mouse brain slices were stained with the antibodies given beside the figures (green) and propidium iodide for cellular counterstaining (red). For preabsorption antibodies were incubated 4 h at RT with a 40 molar excess of the respective recombinant protein before incubation with the brain slices. Bound antibodies were detected by fluorescently labeled secondary antibodies. [file 1471-2202-7-6-S2.pdf]
